# ETISEQ – an algorithm for automated elution time ion sequencing of concurrently fragmented peptides for mass spectrometry-based proteomics

**DOI:** 10.1186/1471-2105-10-244

**Published:** 2009-08-10

**Authors:** Jason WH Wong, Alexander B Schwahn, Kevin M Downard

**Affiliations:** 1 UNSW Cancer Research Centre, University of New South Wales, Sydney, NSW 2052, Australia; 2 School of Molecular and Microbial Biosciences, University of Sydney, Sydney, NSW, 2006, Australia

## Abstract

**Background:**

Concurrent peptide fragmentation (i.e. shotgun CID, parallel CID or MS^E^) has emerged as an alternative to data-dependent acquisition in generating peptide fragmentation data in LC-MS/MS proteomics experiments. Concurrent peptide fragmentation data acquisition has been shown to be advantageous over data-dependent acquisition by providing greater detection dynamic range and providing more accurate quantitative information. Nevertheless, concurrent peptide fragmentation data acquisition remains to be widely adopted due to the lack of published algorithms designed specifically to process or interpret such data acquired on any mass spectrometer.

**Results:**

An algorithm called Elution Time Ion Sequencing (ETISEQ), has been developed to enable automated conversion of concurrent peptide fragmentation data acquisition data to LC-MS/MS data. ETISEQ generates MS/MS-like spectra based on the correlation of precursor and product ion elution profiles. The performance of ETISEQ is demonstrated using concurrent peptide fragmentation data from tryptic digests of standard proteins and whole influenza virus. It is shown that the number of unique peptides identified from the digests is broadly comparable between ETISEQ processed concurrent peptide fragmentation data and the data-dependent acquired LC-MS/MS data.

**Conclusion:**

The ETISEQ algorithm has been designed for easy integration with existing MS/MS analysis platforms. It is anticipated that it will popularize concurrent peptide fragmentation data acquisition in proteomics laboratories.

## Background

Liquid chromatography (LC) coupled electrospray ionization (ESI)-tandem mass spectrometry (MS/MS) [1] has been one of the essential proteomics enabling technologies [2,3]. While technological improvements are continually being made in chromatography [4], mass spectrometry [5,6] and mass spectra interpretation algorithms [7], the detection of lower abundance proteins or proteolytic peptides in complex mixtures remains an obstacle in most proteomics experiments [8,9]. These dynamic range limitations arise in LC-MS/MS experiments, in part, as a result of the inability to completely resolve all peptide ions by liquid chromatography. The use of multidimensional liquid chromatography, where peptides are resolved using two or more separation principles, can improve the dynamic range of detection [10]. Nevertheless, in complex proteomic samples, multiple peptides are still likely to co-elute.

In order to acquire tandem mass spectra for as many peptide ions as possible, the vast majority of tandem mass spectrometers are able to perform data-dependent acquisition (DDA). Data-dependent acquisition of LC-MS/MS data has been the principal method for collecting peptide fragmentation data for both protein identification and quantification. During this process, a preliminary survey MS scan is acquired to identify the peptide ions that elute into the ion source at any point in time. This is followed by one or a series of MS/MS scans to isolate and dissociate each peptide ion in turn, typically in decreasing order of their ion signal abundance. Exclusion lists can be used to prevent repeated sequencing of highly abundant ions that may limit the chance of sample peptide ions from being sequenced. Lists containing *m/z *values of solvent cluster ions, buffer or other known protein contaminants such as keratin may be also used. Nevertheless, DDA may still overlook low abundance ions.

A second disadvantage of DDA is its inability to accurately quantitate peptides in proteomics mixtures. Quantitative information is derived from the selected ion chromatograms (SICs) generated for each of the peptides from the survey MS scans. As the number of peptides ions subjected to tandem mass spectrometry increases per survey MS scan, there will be fewer MS scans from which to quantitate ions during the course of an LC-MS/MS experiment. Ultimately, this will affect the reliability of comparative protein quantification using isotopic labelling [11] or label-free methods [12].

To overcome limitations of DDA-based experiments, the concept of concurrent peptide fragmentation data acquisition (CDA) has been shown to be both feasible [13-15] and provides excellent reproducibility and peptide coverage [16]. During CDA, each survey MS scan is followed by a MS/MS-like scan in which all peptide ions are concurrently dissociated either within the ion source [17] or the dissociation cell [18]. CDA has been variably termed shotgun CID [13], parallel CID [15] and MS^E ^[16]. The advantage of CDA is that in theory all peptide ions will be fragmented regardless of their signal intensity. Furthermore, since CDA acquires survey MS data every alternate scan, quantitative information can be obtained more reliably in comparison to DDA data.

**Figure 1:**
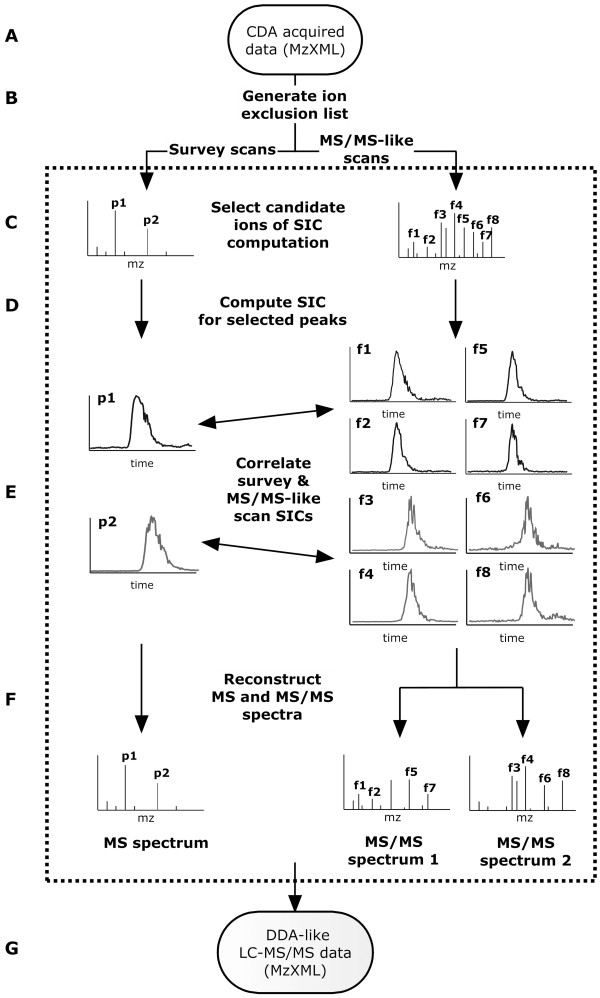
**Schematic diagram of the ETISEQ algorithm**. The major steps of the algorithm are labelled from A to G and explained in the text.

Despite the advantages of CDA over DDA, the method has not been widely adopted. The major reason for this is that, with the exception of a platform specific software package [16], there are no publicly available algorithms designed specifically to process or interpret CDA data acquired on any mass spectrometer. To enable automated analysis, an algorithm termed elution time ion sequencing (ETISEQ) has been designed for processing any CDA data. Using LC elution profiles of precursor and product ions, ETISEQ automatically reconstructs MS/MS-like spectra for peptides which have been concurrently fragmented. In doing so it converts the CDA data into a DDA-like LC-MS/MS dataset (Figure 1). This manuscript describes the design and development of the algorithm. The performance of the algorithm is demonstrated using real CDA data from protein samples with increasing numbers of proteolytic peptides. The output results are compared with DDA data recorded for the same samples.

## Results

### Description and basis of the ETISEQ algorithm

The ETISEQ algorithm will be described stepwise as outlined in Figure 1 from steps *A *to *G*.

### Input file format (A)

ETISEQ has been designed to process CDA data. For the purpose for this algorithm, CDA data is defined as an LC-MS experiment where, throughout the duration of the experiment, survey MS scans are alternated with MS/MS-like scans. These scans will be typically odd and even numbered respectively. To maximize the compatibility of the algorithm with mass spectrometers, ETISEQ accepts CDA data in mzXML format.

### Generation of an ion exclusion list (B)

Since all ions are fragmented during CDA, it is useful to exclude precursor and product ions that are known to be contaminants [19]. ETISEQ has been designed to be able to search for ions that are present in more than 25% of scans. Ions which appear in more than 25% of scans are considered unlikely to be of peptide ions since their detection would be independent of chromatographic separation. Known common LC-MS/MS contaminants [19] can also be manually added to an exclusion list. Ions on the exclusion list will not be considered for all subsequent steps in the algorithm.

### Ion selection from survey and MS/MS-like scans (C)

Ion selection from survey and MS/MS-like scans is an important component of the ETISEQ algorithm since it predetermines the number and quality of reconstructed MS/MS-like spectra. A selected number (*n*) of ions for which a MS/MS spectrum is reconstructed can be defined for each scan. Selected precursor ions can then be excluded from further selection of a given number (*x*) of subsequent scans. The advantage of excluding previously sampled peptide ions is that it allows the reconstruction of MS/MS-like spectra for lower abundance ions while avoiding the generation of redundant MS/MS-like spectra. Selection of ions from MS/MS-like scans is based on a noise threshold (typically > 0.01 of base peak intensity). For both survey and MS/MS-like scans, the ETISEQ algorithm automatically excludes all but the most abundant ion of an isotopic cluster of peaks.

### Computation of SIC for selected peaks (D)

SICs enable the elution time and profiles of precursor and product ions to be correlated. These are computed for all selected peaks from the survey and MS/MS-like scans. SICs are computed over a selected time range (30s either side of the time of detection of a given ion was found to be adequate). Time defined SIC will ensure that related precursor and product ions are correlated accurately since a precursor ion and its product ion will have identical elution times and profiles while other ions of similar or identical *m/z *may exist but will elute at different points in time. Generating time defined SIC also improves the speed of the algorithm as it limits the number of data points that need to be compared.

### Correlation of SICs of ions from survey and MS/MS-like scans (E)

**Figure 2:**
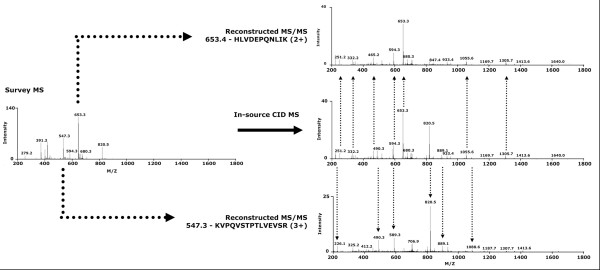
**An example of a reconstructed MS/MS-like spectrum**. The MS/MS-like spectra is reconstructed from an in-source CID MS/MS-like spectrum with two selected peptide ions from the survey scan. The dotted arrows show some of the product ions unique to each reconstructed MS/MS spectrum.

The lineage of product ions to the precursor ions are predicted based on the correlation of their SICs. This is referred to as precursor-product ion association. There are two components which determine whether two SICs are significantly correlated. First, each peptide is expected to have a distinct elution time. The elution profile (i.e. the peak shape in a SIC) is also expected to be unique to each peptide. Figure 2 shows a number of SICs for different ions in the MS/MS-like spectrum.

To compare the elution time of ions, fast Fourier transform (FFT) cross-correlation is used to rapidly determine whether two ions eluted at the same time based on their SICs. The calculation of the cross-correlation function using FFT is a well-known method for measuring correlation and time delay/lag between 1D and 2D signals in signal processing [20]. In proteomics, FFT cross-correlation has been used in a variety of applications, including tandem mass spectra database searching, as in the SEQUEST algorithm [21], and for the alignment of chromatograms and mass spectra [22]. FFT cross-correlation has the attractive property of being computationally efficient for finding the maximal correlation between two data sets where one signal may be shifted relative to another. FFT cross-correlation is superior to comparing peak maxima since the elution profile is taken into consideration and therefore the comparison is more resilient to noise.

For the comparison of the actual shape of the elution profile, the Pearson's correlation coefficient [23] is used to indicate the strength of a linear relationship between two SICs. Pearson's correlation coefficient ranges from +1 to -1. A correlation value of +1 indicates identical positive relationship between two SICs, 0 indicates that two SICs are unrelated and -1 indicates a negative relationship between two SICs. Based on the tested datasets, it was found that an absolute lag of 1 scan and a correlation coefficient of greater than 0.7 was most effective for the correlation of precursor and product ions (data not shown).

Where multiple product ions are correlated to more than one precursor ions, the product ions are assigned to all correlating precursor ions. When a product ion does not match any precursor ion, it is assigned to all precursor ions. For the datasets used for testing ETISEQ, such product ions are typically of low abundance and therefore results in the generation of SICs that contain significant machine noise (data not shown).

### Reconstruction of MS and MS/MS-like spectra (F)

Survey MS scans from CDA data are identical to survey MS scans from DDA data. For each selected precursor ion evident in a survey MS scan, a MS/MS spectrum is reconstructed by elution time correlation of precursor and product ions. Product ions that do not significantly correlate with any precursor ion are included in all corresponding reconstructed MS/MS-like spectra. An example of a reconstructed MS/MS-like spectra generated from an in-source CID spectrum of two peptides is shown in Figure 2.

### Generation of output DDA-like LC-MS/MS data (G)

Steps *C *to *F*, described above, are repeated for each pair of survey MS and MS/MS-like scans. Once all scans have been processed, ETISEQ uses all reconstructed spectra to generate DDA-like LC-MS/MS data in mzXML file format.

### Implementation of the ETISEQ algorithm

The ETISEQ algorithm was implemented using C++ and is compatible with Windows XP/Vista, Linux and Mac OS × operating systems. The standalone executable and a web interface for ETISEQ can be accessed at the following URL address: . The time taken for ETISEQ to run will depend on the parameters, but in general the time required does not exceed 10 minutes. mzXML viewers and inter-conversion tools can be found at the NHLBI Proteomics Center at the Institute for Systems Biology [24].

### Testing

#### Optimization of ETISEQ parameters

**Figure 3:**
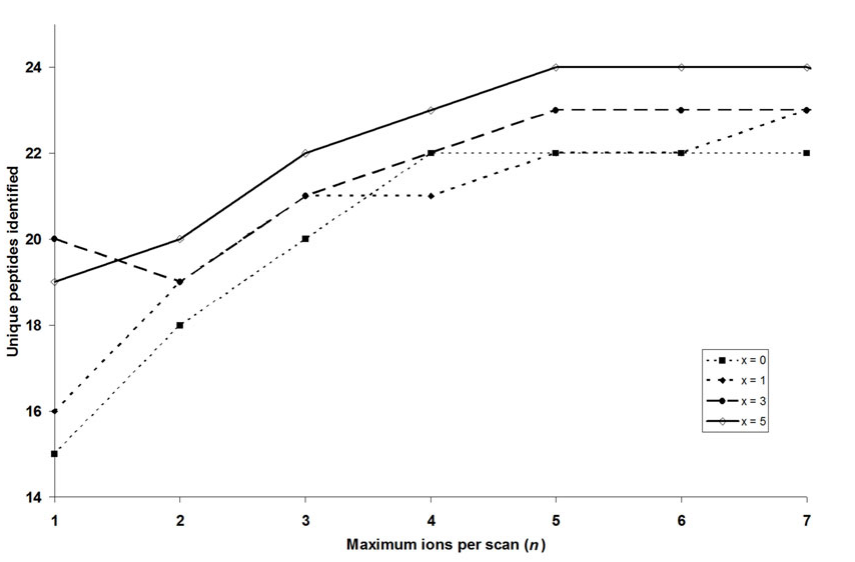
**Parameter optimization**. Relationship between the maximum number of peptide ions selected for each scan, *n *(x-axis), number of subsequent scans to exclude selected ions, *x*, (depicted by different lines and dots) and the number of unique peptides identified for the BSA digest (y-axis).

The key parameters which determine the reconstruction of MS/MS-like spectra are the maximum number of selected precursor ions from the survey MS scans (*n*) and the number of scans (*x*) in which the selected ions are excluded. To optimise the parameters, a range of values were used in combination, and the optimal values were determined based on the number of identified unique peptides for the BSA proteolytic sample. From Figure 3, it can be seen that between 15 and 24 unique peptides could be identified from the reconstructed MS/MS-like spectra. As *n *increases, the number of identified unique peptides generally plateaus above a maximum of 5 ions per selected scan. Increasing *x *from 0 to 5 was found to increase the number of identified unique peptides. The optimal value in this case was to select a maximum of 5 ions per scan and exclude the selected ions for the next 4 scans (i.e. *n *= 5 and *x *= 4). The choice of parameters is likely to vary depending on the chromatography conditions and the complexity of the sample analysed and can be easily adjusted by the user through the ETISEQ web-interface.

Other parameters that that can be adjusted by the user include the addition of the option to exclude contaminants. Furthermore, to increase the speed of the ETISEQ algorithm and to reduce the number of reconstructed MS/MS-like spectra of non-peptide origin, it was found that it is beneficial to predefine the range of scans over which MS/MS reconstruction is performed. This range can usually be estimated by visualization of the CDA data to determine the elution times where the peptide ions are first and last detected.

All remaining parameters described in the previous section such as the SIC time range and correlation coefficient cut-off were found to require less optimisation and are therefore not direct user adjustable through the web-interface. Nevertheless, these can be adjusted in the standalone version of ETISEQ which can be requested from the authors.

#### ETISEQ performance validation

In order to demonstrate the utility of the described algorithm, the numbers of peptides identified for tryptic protein digests of hen egg lysozyme and BSA, as well as a tryptic digest of whole influenza virus were compared using DDA data and CDA data with ETISEQ processing. The parameters used for ETISEQ were 5 selected precursor ions per spectra (*n *= 5) and these were excluded for 4 subsequent scans (*x *= 4) as previously determined to be optimal. DDA-like LC-MS/MS data were also generated from CDA data without precursor-product ion correlation processing. This was necessary to demonstrate the benefit of ion correlation on automated MS/MS spectra interpretation.

Table 1 shows that the DDA data resulted in the highest number of unique peptides for identified lysozyme and the virus digest (8 and 20 unique peptides respectively), while CDA data processed with ETISEQ identified most for BSA (24 unique peptides – for full list of peptides [see Additional file 1]). In all cases, CDA without correlation processing resulted in the least number of identified unique peptides. Based on the overall peptide coverage of the virus digest (Table 2), it can be seen that the some of the identified peptides are different between DDA and CDA acquired data.

**Table 1:** Total number of unique peptides identified using MS/MS spectra interpretation algorithms for lysozyme, BSA and virus digests.

	**Unique peptides^**		
	**Lysozyme**	**BSA**	**Virus**
**DDA**	8 (60.5%)	21 (36.2%)	20
**CDA – ETISEQ processed**	7 (52.4%)	24 (32.3%)	15
**CDA – no correlation processing**	6 (52.4%)	19 (27.0%)	10

^ For lysozyme and BSA, the respective sequence coverage is shown in brackets. The data are generated by data dependent LC-MS/MS and CDA with and without ETISEQ processing. [See Additional file 1] for protein/peptide list.

**Table 2:** Summary of proteins and identified unique peptides for the tryptic digest of the influenza virus preparation.

		**Peptides^**	
**Accession**	**Description**	**DDA**	**CDA – ETISEQ**
P03466	Nucleoprotein – Influenza A	MVLSAFDER (2+)	-
	(Puerto Rico/8/1934 H1N1)	ASAGQISIQPTFSVQR (2+)	ASAGQISIQPTFSVQR (2/3+)
		TTIMAAFNGNTEGR (2+)	-
		MMESARPEDVSFQGR (3+)	MMESARPEDVSFQGR (2/3+)
		ESARPEDVSFQGR (2+)	ESARPEDVSFQGR (2+)
		-	SARPEDVSFQGR (2+)
		[10.8%]	[6.2%]
gi|33622382	Hemagglutinin – Influenza A	EVLVLWGVHHPPNIGNQR (3+)	EVLVLWGVHHPPNIGNQR (3+)
	(New Caledonia/20/1999 H1N1)	ALYHTENAYVSVVSSHYSR (3+)	ALYHTENAYVSVVSSHYSR (3+)
		-	HPPNIGNQR (2+)
		[6.6%]	[6.6%]
gi|22859465	Matrix protein 1 – Influenza A	QMVTTTNPLIR (2+)	QMVTTTNPLIR (2+)
	(New Caledonia/20/1999 H1N1)		
		[5.2%]	[5.2%]
P01012	Ovalbumin – Gallus gallus	GGLEPINFQTAADQAR (2+)	GGLEPINFQTAADQAR (2+)
		NVLQPSSVDSQTAMVLVNAIVFK (3+)	-
		AFKDEDTQAMPFR (3+)	-
		LTEWTSSNVMEER (2+)	LTEWTSSNVMEER (2+)
		ISQAVHAAHAEINEAGR (3+)	ISQAVHAAHAEINEAGR (3+)
		[21.2%]	[11.9%]
P02789	Ovotransferrin precursor – Gallus gallus	VEDIWSFLSK (2+)	-
		YFGYTGALR (2+)	YFGYTGALR (2+)
		GDVAFIQHSTVEENTGGK (3+)	-
		ECNLAEVPTHAVVVRPEK (3+)	-
		FMMFESQNK (2+)	-
		[9.1%]	[1.3%]
P01014	Ovalbumin-related protein Y – Gallus gallus	YNPTNAILFFGR (2+)	YNPTNAILFFGR (2+)
		-	PTNAILFFGR (2+)
		HSLELEEFR (2+)	HSLELEEFR (2+)
		[5.4%]	[5,4%]

^The sequence coverage of each protein is shown in square brackets. The data are acquired by DDA and CDA with ETISEQ processing. Note that the virus was cultured in 10–14 day old embryonic hen eggs (*Gallus gallus*) and therefore chicken proteins are expected.

**Figure 4:**
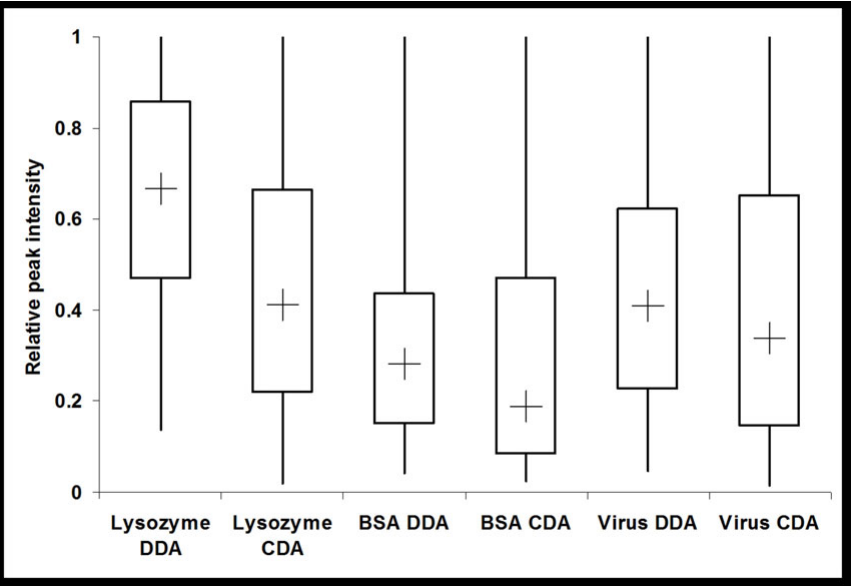
**Distribution of relative abundances of peptides sampled**. The box-plot shows the distribution of the relative intensity of precursor ions of MS/MS (DDA) and reconstructed MS/MS-like (CDA) spectra for the lysozyme, BSA and whole virus preparation tryptic digest. The lines indicated the maximum and minimum relative abundances, the box edges indicate the first and third quartile of relative abundances and the cross (+) indicates the median relative abundance.

The distribution of the relative abundance of precursor ions that were correctly identified was also examined. For all three samples analysed it was found that, in comparison to DDA, CDA resulted in the identification of precursor ions with a broader range of relative intensities as well as lower median and minimum relative intensities (Figure 4).

## Discussion

The results demonstrate that CDA generated data can be readily used for protein identification when processed using the described algorithm. While proteins can still be identified from CDA data without correlation processing of precursor and product ion lineage, the results show that it is advantageous to do so (Table 1). This is expected as the presence of unrelated product ions in MS/MS spectra will lead to erroneous identifications by MS/MS spectra interpretation algorithms [25]. The peptide identification results for ETISEQ generated DDA-like LC-MS/MS data are broadly comparable to DDA data (Table 1). For the whole influenza virus digest, there were a number of identified peptides that were unique to CDA data despite the fact that less unique peptides were identified for the reconstructed LC-MS/MS data in comparison to DDA data (Table 2). This is expected as, unlike DDA, CDA experiments will sample peptides independent of its abundance and generate peptide fragment data of all peptides. Indeed, Figure 4 shows that the distribution of CDA data sampled was more tailed with a lower median compared to DDA data, indicating that more low abundance ions were sequenced in CDA experiments.

For the virus digest (Table 2). It should be noted that peptides unique to CDA were in all cases a subsequence of the tryptic peptides detected in DDA. This may be due to the generation of in-source fragments during precursor ions scans resulting from incomplete evacuation of the cooling gas following a product ion scan. The acquisition of CDA data by true MS/MS should alleviate this problem. Nevertheless, the ability of ETISEQ to generate MS/MS spectra for such ions demonstrates the utility of the software.

In part, the difference in the identified peptides may be explained by the differing product ions that are generated during CDA and single peptide MS/MS data. In the experiments described, concurrent peptide fragmentation was achieved in the ion source where ions are accelerated across the source under atmospheric pressure. For DDA data, the dissociation of peptide ions occurs within a collision cell or chamber. While both methods generally yield b- and y- type product ions [26], in-source CID produces product ions under a different environment in the presence of residual solvent and other atmospheric gases. CDA can be performed on single mass analyser instruments (without tandem MS capabilities). Furthermore, the detection efficiency for fragment and precursor ions can be enhanced since both have shorter paths to the detector. It is important to realise that in the case of CDA experiments, the possibility of performing true tandem experiment is not excluded. This may be achieved by adjustment of ion focusing to allow for the transmission of all precursor ions (at once) into a collision cell [15]. In this instance, and in in-source CID experiments, different multiply charged ions of a common peptide are collectively fragmented thereby increasing the overall product ion intensities and improving the signal-to-noise ratio.

It is anticipated that CDA along with ETISEQ processing, will be most advantageous for quantitative experiments that make use of affinity enrichment and isotope labelling such as isotope-coded affinity tags [11]. In comparison to DDA it can be expected that CDA will produce more SIC data points for peptide ions since survey MS scans are always performed every alternate scan. In contrast to CDA, it is common in DDA that survey MS scans are only acquired every 3–4 scans. The same quantitative advantage can be expected for label-free MS-based experiments that make use of SICs [27].

## Conclusion

The ETISEQ algorithm enables automated processing of CDA data for protein identification. The algorithm has the ability to discover precursor-product ion lineages in order to reconstruct MS/MS-like spectra. It enables the conversion of CDA data to DDA-like LC-MS/MS data. The ETISEQ algorithm has been designed to input and output the data in mzXML file format to ensure maximum compatibility with data from different MS instruments and downstream MS/MS analysis platforms.

A comparison of database search results from DDA and ETISEQ processed CDA data for tryptic digest of two standard proteins and whole virus demonstrate that results from both strategies are comparable in their performance. Importantly, the use of the ETISEQ algorithm on CDA data significantly increased the number of unique peptides identified. It was also found that CDA samples a wide abundance of peptide ions in comparison to DDA. With further optimization of the CDA peptide fragmentation process, it can be anticipated that ETISEQ will be of great value to enable CDA where quantification is required.

## Methods

### Materials

All standard chemicals were purchased from commercial sources and were all of analytical grade, unless stated otherwise. Protease inhibitor cocktail and modified trypsin (sequence grade) were obtained from Promega (Madison, WI, USA). Influenza strain A/New Caledonia/20/99 IVR116 (H1N1) was obtained from Advanced ImmunoChemicals Inc. (Long Beach, California USA) as an inactivated virus preparation from the allantoic fluid of 10–11 day old embryonated eggs. All other chemicals and reagents were obtained from Sigma-Aldrich (Sydney, Australia).

### Proteolytic sample preparation

In-solution tryptic digestion of the proteins and virus was performed. Briefly, standard proteins lysozyme and bovine serum albumin (BSA) were adjusted to a concentration of 1 mg/mL in 50 mM NH_4_HCO_3_, pH 7. Samples were reduced with DTT, and treated with iodoacetamide to alkylate cysteines and then incubated with trypsin at 37°C overnight. For the influenza virus, 50 μg of the virus (corresponding to 38.5 μL of the virus suspension) were concentrated to near dryness in a vacuum concentrator, resuspended in 50 μL digestion buffer (50 mM NH_4_HCO_3_, 10% Acetonitrile, 2 mM DTT) and incubated at 37°C for 4 h prior to the addition of trypsin and overnight digestion. All digestions were performed at a 1:100 trypsin to protein ratio.

### Liquid chromatography – Mass spectrometry

Proteolytic samples were analysed by LC-MS, using a nanoflow HPLC system (Agilent 1100, Agilent Technologies) coupled with a quadrapole time-of-flight mass spectrometer (QStar XL Hybrid, Applied Biosystems) equipped with a nanospray source. Samples were loaded onto a reverse phase C_18 _column (Zorbax Eclipse XDB, 5 μm, 0.3 × 150 mm) and eluted into the ion source at a flow rate of 0.8 μL/min using a mobile phase of H_2_O (Buffer A)/acetonitrile (Buffer B). Specifically, the gradient conditions were as follows: 95:5 (A:B) at 0 min, changing linearly to 90:10 (A:B) at 5 min, changing linearly to 50:50 (A:B) at 40 min, changing linearly to 30:70 (A:B) at 45 min, changing linearly to 95:5 (A:B) at 50 min and then unchanged until the completion of the run at 60 min.

To acquire CDA data, an instrument method was created to acquire alternate survey MS scans followed by in-source induced fragmentation to create MS/MS-like spectra. The alternation was set for the entire duration of the LC-MS experiment. To induce in-source fragmentation of peptides, the declustering potential (potential between the orifice plate and the skimmer) was increased from a typical value of 50 V to 155 V, while the collision cooling gas pressure was also increased from the typical value of 3 to 8 (arbitrary units). For DDA, the two most abundant ions for each survey MS scan were selected for subsequent MS/MS analysis. Once a MS/MS spectrum was acquired for a given ion, it is excluded from a further MS/MS experiment for the next 30 seconds. For all downstream data analysis, data files in .wiff format were centroided and converted to mzXML format using the MzWiff software [28].

It should be noted that the acquisition of CDA data by passing all peptides into a collision cell was attempted. However, it was found that this could not be achieved efficiently on the QStar XL Hybrid used in this experiment. As a result, in-source induced dissociation was performed as an alternative.

### Data analysis

All CDA and DDA LC-MS/MS data in mzXML file format were analysed using the InsPecT (version 20080404) [29], X!Tandem (version 2008.02.01) [30] and OMSSA (version 2.1.1) [31] algorithms with a custom database which includes all *Gallus gallus *and influenza virus proteins, BSA, trypsin and human keratin proteins within the UniprotKB/Swiss-Prot database (Release 56.0, 22-Jul-2008). Influenza strain A/New Caledonia/20/99 (H1N1) proteins retrieved from the Influenza Virus Resource provided by the National Center for Biotechnology Information were also included. Multiple search algorithms were used to reduce the likelihood of false positive identifications as well as to maximise the number of peptides identified. Database search parameters were set at 2.5 Da and 0.5 Da for precursor and product ion error tolerance respectively. Semi-tryptic digestion allowing for 2 missed cleavages was used. Carbamidomethyl cysteine, and the possibility of methionine oxidation and pyroglutamate formation, were specified. Peptide identification acceptance cut-offs values of p-value < 0.025 for InsPect, e-value < 0.01 for X!Tandem and e-value < 0.025 for OMSSA were used. The cut-offs are determined from an observed 1% false positive rate (FDR) using a random decoy database [32]. FDR is defined as, False Positives/(True Positives+False Positives). Specifically, cut-offs for each algorithm was selected such that no DDA or CDA datasets used in this analysis had a FDR of greater than 1%.

## Availability and requirements

**Project name: **ETISEQ

**Project home page: **

**Operating system: **Windows, Linux

Programming language: C++

**License: **Free for non-commercial use. Source code available upon request.

## Authors' contributions

JWHW conceived the study, carried out the mass spectrometry experiments, developed and implemented the algorithm and wrote the manuscript. ABS carried out the mass spectrometry experiments, prepared the virus digest and helped draft the manuscript. KMD participated in its design and coordination and helped to draft the manuscript. All authors read and approved the final manuscript.

## Supplementary Material

Additional file 1 **List of proteins/peptides identified**. Lists of proteins/peptides identified for the tryptic digests of Chicken Lysozyme, Bovine Serum Albumin and Virus preparation from data acquired by DDA, CDA with ETISEQ processing and CDA without ETISEQ processing. Click here for file
